# Supplementary material for: Coding with transient trajectories in recurrent neural networks
Source: PLoS Comput Biol. 2020 Feb 13;16(2):e1007655. doi: 10.1371/journal.pcbi.1007655 (PMC7043794; doi:10.1371/journal.pcbi.1007655)
Supplement: S1 Text — (PDF) [file pcbi.1007655.s001.pdf]

Coding with transient trajectories in recurrent neural networks

Giulio Bondanelli [*1], Srdjan Ostojic [1],

1 Laboratoire de Neurosciences Cognitives et Computationelles, Département d'Études Cognitives, École Normale Supérieure, INSERM U960, PSL University, Paris, France

*giulio.bondanelli@ens.fr

Supporting information

S1 Text

By the definition of singular value decomposition we can express the k-th singular value of $\mathbf{P}_t$ as $\sigma_k = \mathbf{L}_k^T \mathbf{P}_t \mathbf{R}_k$ and the squared k-th singular values as $\sigma_k^2 = \mathbf{L}_k^T \mathbf{P}_t \mathbf{P}_t^T \mathbf{L}_k$. By differentiating σ_k^2 we can write

$$\frac{\mathrm{d}\sigma_k^2}{\mathrm{d}t} = \dot{\mathbf{L}}_k^T \mathbf{P}_t \mathbf{P}_t^T \mathbf{L}_k + \mathbf{L}_k^T \dot{\mathbf{P}} \mathbf{P}_t^T \mathbf{L}_k + \mathbf{L}_k^T \mathbf{P}_t \dot{\mathbf{P}}^T \mathbf{L}_k + \mathbf{L}_k^T \mathbf{P}_t \mathbf{P}_t^T \dot{\mathbf{L}}_k \tag{96}$$

The first term can be expressed as $\dot{\mathbf{L}}_k^T \mathbf{P}_t \mathbf{P}_t^T \mathbf{L}_k = \dot{\mathbf{L}}_k^T \mathbf{L}_k \sigma_k^2$. Since the right singular vector $\mathbf{L}_k$ has unit norm, the scalar product between $\mathbf{L}_k$ and its derivative $\dot{\mathbf{L}}_k$ is equal to zero. The same holds for the last term on the right hand side $\mathbf{L}_k^T \mathbf{P}_t \mathbf{P}_t^T \dot{\mathbf{L}}_k$. Thus, we can rewrite Eq. (96) as

$$\begin{aligned} \frac{\mathrm{d}\sigma_k^2}{\mathrm{d}t} &= \mathbf{L}_k^T \mathbf{P}_t (\mathbf{J} + \mathbf{J}^T - 2\mathbf{I}) \mathbf{P}_t^T \mathbf{L}_k \\ &= 2\sigma_k^2 \, \mathbf{R}_k^T (\mathbf{J}_S - \mathbf{I}) \mathbf{R}_k \end{aligned} \tag{97}$$

where the last equality follows from $\mathbf{L}_k^T \mathbf{P}_t = \mathbf{L}_k^T \sum_j \sigma_j \mathbf{L}_j \mathbf{R}_j^T = \sigma_k \mathbf{R}_k^T$. By definition, at the optimal time t^*, the derivative of the largest singular value $\sigma_1(\mathbf{P}_t)$ vanishes. Since σ_1^2 is a monotonic function of σ_1, at time t^* also the derivative in Eq. (97) vanishes. Thus, the optimal initial condition $\mathbf{R}_1^{(t^*)}$ satisfies Eq. (19).

Following the same steps we can obtain the same equation in terms of the left singular values of the propagator $\mathbf{L}_k$:

$$\frac{\mathrm{d}\sigma_k^2}{\mathrm{d}t} = 2\sigma_k^2 \, \mathbf{L}_k^T (\mathbf{J}_S - \mathbf{I}) \mathbf{L}_k \tag{98}$$
