# Supplementary material for: Coding with transient trajectories in recurrent neural networks
Source: PLoS Comput Biol. 2020 Feb 13;16(2):e1007655. doi: 10.1371/journal.pcbi.1007655 (PMC7043794; doi:10.1371/journal.pcbi.1007655)
Supplement: S2 Text — (PDF) [file pcbi.1007655.s002.pdf]

Coding with transient trajectories in recurrent neural networks

Giulio Bondanelli [*1], Srdjan Ostojic [1],

1 Laboratoire de Neurosciences Cognitives et Computationelles, Département d'Études Cognitives, École Normale Supérieure, INSERM U960, PSL University, Paris, France

*giulio.bondanelli@ens.fr

Supporting information

S2 Text

For any N-dimensional matrix $\mathbf{A}$, we can express its exponential as

$$\exp(t\mathbf{A}) = \sum_{j=0}^{N-1} x_j(t)\mathbf{A}^j, \tag{99}$$

where the $x_j(t)$ $(0 \le j \le N-1)$ are the N solutions of the N-th order differential equation

$$x^{(N)} + c_{N-1}x^{(N-1)} + ... + c_1 x^{(1)} + c_0 x = 0 \tag{100}$$

with the set of N initial conditions $x_j^{(l)}(0) = \delta_{jl}$ [1]

$$\begin{cases} x_0^{(0)} = 1 \\ x_0^{(1)} = 0 \\ \vdots \\ x_0^{(N-1)} = 0 \end{cases}, \quad \begin{cases} x_1^{(0)} = 0 \\ x_1^{(1)} = 1 \\ \vdots \\ x_1^{(N-1)} = 0 \end{cases}, \quad \cdots \quad \begin{cases} x_{N-1}^{(0)} = 0 \\ x_{N-1}^{(1)} = 0 \\ \vdots \\ x_{N-1}^{(N-1)} = 1 \end{cases} \tag{101}$$

with $0 \le j \le N-1$ and $0 \le l \le N-1$. $x_j^{(n)}$ denotes the n-th derivative of the solution $x_j(t)$, while the numbers c_i are the coefficients in the expression of the characteristic polynomial of $\mathbf{A}$

$$\mathrm{Det}(\lambda\mathbf{I} - \mathbf{A}) = \lambda^N + c_{N-1}\lambda^{N-1} + ... + c_1\lambda + c_0. \tag{102}$$

References

[1] Leonard IE. The matrix exponential. SIAM Review. 1996;38(3):507–512.
