# Supplementary material for: Coding with transient trajectories in recurrent neural networks
Source: PLoS Comput Biol. 2020 Feb 13;16(2):e1007655. doi: 10.1371/journal.pcbi.1007655 (PMC7043794; doi:10.1371/journal.pcbi.1007655)
Supplement: S3 Text — (PDF) [file pcbi.1007655.s003.pdf]

Coding with transient trajectories in recurrent neural networks

Giulio Bondanelli *1, Srdjan Ostojic 1,

1 Laboratoire de Neurosciences Cognitives et Computationelles, Département d'Études Cognitives, École Normale Supérieure, INSERM U960, PSL University, Paris, France

*giulio.bondanelli@ens.fr

Supporting information

S3 Text

We express the eigenvalues of $\mathbf{J}_S$ as

$$\begin{cases} \lambda_S^+ = \dfrac{\lambda_S^+ + \lambda_S^-}{2} + \dfrac{\lambda_S^+ - \lambda_S^-}{2} \\ \lambda_S^- = \dfrac{\lambda_S^+ + \lambda_S^-}{2} - \dfrac{\lambda_S^+ - \lambda_S^-}{2} \end{cases} \tag{103}$$

and split the propagator into the sum of two terms:

$$\begin{aligned} \exp(t\mathbf{J}') &= \begin{pmatrix} x_0(t) + \frac{\lambda_S^+ + \lambda_S^-}{2} x_1(t) & x_1(t)\Delta \\ -x_1(t)\Delta & x_0(t) + \frac{\lambda_S^+ + \lambda_S^-}{2} x_1(t) \end{pmatrix} + \begin{pmatrix} \frac{\lambda_S^+ - \lambda_S^-}{2} x_1(t) & 0 \\ 0 & -\frac{\lambda_S^+ - \lambda_S^-}{2} x_1(t) \end{pmatrix} \\ &= \begin{pmatrix} E(t) & H(t) \\ -H(t) & E(t) \end{pmatrix} + \begin{pmatrix} F(t) & G(t) \\ G(t) & -F(t) \end{pmatrix}, \end{aligned} \tag{104}$$

where the time-dependent functions E, F, G, H are given by

$$\begin{cases} E(t) = x_0(t) + x_1(t)(\lambda_S^+ + \lambda_S^-)/2 \\ F(t) = x_1(t)(\lambda_S^+ - \lambda_S^-)/2 \\ G(t) = 0 \\ H(t) = x_1(t)\Delta. \end{cases} \tag{105}$$

If we write the SVD of $\mathbf{P}'_t$ as

$$e^{t(\mathbf{J}'-\mathbf{I})} = \mathbf{L}\mathbf{\Sigma}\mathbf{R}^T = \begin{pmatrix} \cos\beta & \sin\beta \\ -\sin\beta & \cos\beta \end{pmatrix} \begin{pmatrix} \sigma_1 & 0 \\ 0 & \sigma_2 \end{pmatrix} \begin{pmatrix} \cos\gamma & \sin\gamma \\ -\sin\gamma & \cos\gamma \end{pmatrix} \tag{106}$$

we can express the time-dependent parameters $\sigma_1(t), \sigma_2(t), \beta(t), \gamma(t)$ ($\sigma_1 \geq \sigma_2$) as functions of $E(t), F(t), G(t), H(t)$ (Eq. (105)):

$$\begin{aligned} \sigma_1(t) &= e^{-t}\sqrt{E^2 + H^2} + e^{-t}\sqrt{F^2 + G^2} \\ \sigma_2(t) &= e^{-t}\sqrt{E^2 + H^2} - e^{-t}\sqrt{F^2 + G^2} \\ 2\gamma(t) &= \text{atan}(H/E) + \text{atan}(G/F) \\ 2\beta(t) &= \text{atan}(H/E) - \text{atan}(G/F). \end{aligned} \tag{107}$$
