# Supplementary material for: Coding with transient trajectories in recurrent neural networks
Source: PLoS Comput Biol. 2020 Feb 13;16(2):e1007655. doi: 10.1371/journal.pcbi.1007655 (PMC7043794; doi:10.1371/journal.pcbi.1007655)
Supplement: S4 Text — (PDF) [file pcbi.1007655.s004.pdf]

Coding with transient trajectories in recurrent neural networks

Giulio Bondanelli [*1], Srdjan Ostojic [1],

1 Laboratoire de Neurosciences Cognitives et Computationelles, Département d'Études Cognitives, École Normale Supérieure, INSERM U960, PSL University, Paris, France

*giulio.bondanelli@ens.fr

Supporting information

S4 Text

Since the trace is a linear operator and the trace of $\mathbf{J}$ is equal to the trace of $\mathbf{J}^T$, we can express the trace of $\mathbf{J}_S$ as

$$\mathrm{Tr}\mathbf{J}_S = \frac{\mathrm{Tr}(\mathbf{J}+\mathbf{J}^T)}{2} = \lambda_S^+ + \lambda_S^- = \lambda. \tag{108}$$

The determinant $\mathrm{Det}'\mathbf{J}_S$ is simply given by the product of the eigenvalues of $\mathbf{J}_S$:

$$2\mathrm{Det}'\mathbf{J}_S = 2\lambda_S^+\lambda_S^- = (\lambda_S^+ + \lambda_S^-)^2 - {\lambda_S^+}^2 - {\lambda_S^-}^2 = (\mathrm{Tr}\mathbf{J}_S)^2 - \mathrm{Tr}(\mathbf{J}_S^2). \tag{109}$$

The last equality in Eq. (109) follows from the fact that the trace of the square of a matrix is the sum of its squared eigenvalues. Computing $\mathbf{J}_S^2$ yields

$$4\mathbf{J}_S^2 = \Delta^2(\mathbf{u}\mathbf{v}^T + \mathbf{v}\mathbf{u}^T)(\mathbf{u}\mathbf{v}^T + \mathbf{v}\mathbf{u}^T) = 2\lambda\mathbf{J}_S + \Delta^2\mathbf{u}\mathbf{u}^T + \Delta^2\mathbf{v}\mathbf{v}^T. \tag{110}$$

Thus $\mathrm{Tr}(\mathbf{J}_S^2) = (\lambda^2+\Delta^2)/2$ and $2\mathrm{Det}'\mathbf{J}_S = (\lambda^2-\Delta^2)/2$. It follows that the eigenvalues of $\mathbf{J}_S$ (Eq. 43) are given by $\lambda_S^{\pm} = (\lambda \pm \Delta)/2$.
