# Supplementary material for: Coding with transient trajectories in recurrent neural networks
Source: PLoS Comput Biol. 2020 Feb 13;16(2):e1007655. doi: 10.1371/journal.pcbi.1007655 (PMC7043794; doi:10.1371/journal.pcbi.1007655)
Supplement: S5 Text — (PDF) [file pcbi.1007655.s005.pdf]

Coding with transient trajectories in recurrent neural networks

Giulio Bondanelli *[1], Srdjan Ostojic [1],

1 Laboratoire de Neurosciences Cognitives et Computationelles, Département d'Études Cognitives, École Normale Supérieure, INSERM U960, PSL University, Paris, France

*giulio.bondanelli@ens.fr

Supporting information

S5 Text

To compute the singular values of the propagator for the unit-rank system, it is convenient to express the matrix

$$e^{2t}\,\mathbf{P}_t^T\mathbf{P}_t = \mathbf{I} + 2\alpha(t;\lambda)\mathbf{J}_S + \Delta^2\alpha^2(t;\lambda)\mathbf{v}\mathbf{v}^T \tag{111}$$

in the basis of the eigenvectors of $\mathbf{J}_S$. While the second term on the right hand side yields a diagonal contribution proportional to $\mathbf{diag}(\lambda_S^+, \lambda_S^-)$, for the third term we obtain

$$\mathbf{V}_S^T\mathbf{v}\mathbf{v}^T\mathbf{V}_S = \frac{1}{2}\begin{pmatrix} \rho+1 & -\sqrt{1-\rho^2} \\ -\sqrt{1-\rho^2} & 1-\rho \end{pmatrix}. \tag{112}$$

The squared singular values of the propagator $\mathbf{P}_t$ are therefore the eigenvalues of the matrix

$$e^{2t}\,\mathbf{P}_t^T\mathbf{P}_t = \begin{pmatrix} (\rho+1)(a+b)+1 & -b\sqrt{1-\rho^2} \\ -b\sqrt{1-\rho^2} & (\rho-1)(a-b)+1 \end{pmatrix}, \tag{113}$$

where we defined $a = \Delta\alpha(t;\lambda)$ and $2b = \Delta^2\alpha^2(t;\lambda)$. Thus, we have

$$e^{2t}\sigma_{1,2}^2(\mathbf{P}_t) = 1 + a\rho + b \pm \sqrt{a^2+b^2+2ab\rho}, \tag{114}$$

where $\sigma_1(\mathbf{P}_t) > \sigma_2(\mathbf{P}_t)$. Therefore, expanding Eq. (114) we obtain the two singular values $\sigma^\pm$ of $\mathbf{P}_t$:

$$e^{2t}\sigma_{1,2}^2(\mathbf{P}_t) = 1 + \Delta\alpha(t)\rho + \frac{\Delta^2\alpha(t)^2}{2} \pm \Delta\alpha(t)\sqrt{\Delta\alpha(t)\rho + \frac{\Delta^2\alpha(t)^2}{4} + 1}. \tag{115}$$
