# Supplementary material for: Coding with transient trajectories in recurrent neural networks
Source: PLoS Comput Biol. 2020 Feb 13;16(2):e1007655. doi: 10.1371/journal.pcbi.1007655 (PMC7043794; doi:10.1371/journal.pcbi.1007655)
Supplement: S6 Text — (PDF) [file pcbi.1007655.s006.pdf]

Coding with transient trajectories in recurrent neural networks

Giulio Bondanelli [*1], Srdjan Ostojic [1],

1 Laboratoire de Neurosciences Cognitives et Computationelles, Département d'Études Cognitives, École Normale Supérieure, INSERM U960, PSL University, Paris, France

*giulio.bondanelli@ens.fr

Supporting information

S6 Text

An interesting application of the results that we found for the unit-rank connectivity is the system composed of one excitatory and one inhibitory populations. The interactions between the two populations are described by the connectivity matrix

$$\mathbf{J} = \begin{pmatrix} w & -kw \\ w & -kw \end{pmatrix}, \tag{116}$$

where w is the excitatory weight and k represents the relative strength of inhibition with respect to the strength of excitation. We consider the regime in which inhibition is stronger than excitation, i.e. $k > 1$. Since $\mathbf{J}$ has unit rank, we can express it in the form given by Eq. (41), where

$$\begin{cases} \Delta = w\sqrt{2(1+k^2)} \\ \mathbf{u} = \dfrac{(1,1)^T}{\sqrt{2}} \\ \mathbf{v} = \dfrac{(1,-k)^T}{\sqrt{1+k^2}}. \end{cases} \tag{117}$$

Therefore, $\mathbf{J}$ has only one eigenvalue equal to

$$\lambda = w(1-k) \tag{118}$$

and one zero eigenvalue, while the correlation between the structure vectors $\mathbf{u}$ and $\mathbf{v}$ is given by

$$\rho = \frac{1-k}{\sqrt{2(1+k^2)}}. \tag{119}$$

Note that the correlation ρ depends only on k. For simplicity, we assume that k is fixed and slightly larger than unity:

$$k = 1 + \epsilon_k. \tag{120}$$

Thus, for $\epsilon_k \ll 1$, the parameters of the network are given by

$$\begin{cases} \Delta = 2w\left(1 + \dfrac{\epsilon_k}{2}\right) \\ \mathbf{u} = \dfrac{(1,1)^T}{\sqrt{2}} \\ \mathbf{v} = \dfrac{1}{\sqrt{2}}\begin{pmatrix} 1 \\ -1 \end{pmatrix} - \dfrac{\epsilon_k}{2}\mathbf{u} \\ \rho = -\dfrac{\epsilon_k}{2}. \end{cases} \tag{121}$$

Computing the symmetric part of the connectivity $\mathbf{J}$ yields

$$\mathbf{J}_S = \begin{pmatrix} w & w(1-k)/2 \\ w(1-k)/2 & -kw \end{pmatrix}, \tag{122}$$

which has eigenvalues

$$\lambda_S^{\pm} = \frac{w}{2}(1-k) \pm \frac{w}{2}\sqrt{2(1+k^2)}. \tag{123}$$

The condition $\lambda_S^{+}(w,k) > 1$ determines the region of the parameters w and k where transient amplification occurs. In the inhibition-dominated regime the system exhibits amplification if the excitatory strength w is approximately larger than one, when k is larger than but close to unity. In fact, if k is given by Eq. (120), we can write

$$\lambda_S^{+} \simeq w\left(1 + \frac{\epsilon_k^2}{4}\right) \tag{124}$$

so that the transient regime is defined by the condition

$$w \gtrsim 1 - \frac{\epsilon_k^2}{4} \tag{125}$$

We note that the inhibition-dominated regime $k > 1$ is not necessary to achieve transient amplification, since a stable system can be amplified for $k > 1$ or $0 < k < 1$. However, for fixed $0 < k < 1$ the stability constraint $k > 1 - 1/w$ limits the parameter region where stable amplification occurs (Fig. 2). For strongly amplified systems ($w \gg 1$) this region is defined by the stability boundary $k > 1 - 1/w \simeq 1$, which approximately corresponds to the inhibition-dominated unit-rank EI networks.

In the regime of strong amplification (Eq. 55), we can compute the optimal initial condition $\mathbf{R}_1^*$ and the corresponding readout vector $\mathbf{L}_1^*$. If Eq. (120) holds, the strong amplification condition is simply given by $w \gg 1$. Using Eq. (69) we find

$$\begin{cases} \mathbf{R}_1^* = \frac{1}{\sqrt{2}}\begin{pmatrix} 1 \\ -1 \end{pmatrix} + \frac{1}{\sqrt{2}}\left(\frac{1}{2w} - \frac{\epsilon_k}{4}\right)\begin{pmatrix} 1 \\ 1 \end{pmatrix} \\ \mathbf{L}_1^* = \frac{1}{\sqrt{2}}\begin{pmatrix} 1 \\ 1 \end{pmatrix} + \frac{1}{\sqrt{2}}\left(\frac{1}{2w} + \frac{\epsilon_k}{4}\right)\begin{pmatrix} 1 \\ -1 \end{pmatrix}. \end{cases} \tag{126}$$

We find that the optimal initial condition and the optimal readout are aligned respectively with the modes $(1,-1)$ and $(1,1)$. These modes correspond to the patterns of differential and equal firing of the excitatory and inhibitory units, respectively called the difference and sum modes in [1]. The analysis of balanced amplification in [1] explicitly focuses on the dynamics of the sum and difference modes. Here, by studying the amplification of the norm $||\mathbf{r}(t)||$, we show that these modes emerge as the optimal initial condition and the optimal readout, in the limit of strong amplification ($w \gg 1$) and in the inhibition-dominated regime (with $k = 1 + \epsilon_k$). Consistent with [1], our theory shows that under these assumptions a difference in the firing of the E and I units drives strong changes in the pattern of common activation of E and I neurons.

References

[1] Murphy BK, Miller KD. Balanced amplification: a new mechanism of selective amplification of neural activity patterns. Neuron. 2009;61(4):635–648.

[2] Leonard IE. The matrix exponential. SIAM Review. 1996;38(3):507–512.
