# Supplementary material for: Coding with transient trajectories in recurrent neural networks
Source: PLoS Comput Biol. 2020 Feb 13;16(2):e1007655. doi: 10.1371/journal.pcbi.1007655 (PMC7043794; doi:10.1371/journal.pcbi.1007655)
Supplement: S7 Text — (PDF) [file pcbi.1007655.s007.pdf]

# Coding with transient trajectories in recurrent neural networks

Giulio Bondanelli [*1], Srdjan Ostojic [1],

**1** Laboratoire de Neurosciences Cognitives et Computationelles, Département d'Études Cognitives, École Normale Supérieure, INSERM U960, PSL University, Paris, France

*giulio.bondanelli@ens.fr

## Supporting information

### S7 Text

The expression of the propagator and the mean activity are given by

$$\begin{cases} \left[e^{t(\Delta \mathbf{u}\mathbf{v}^T - \mathbf{I})}\right]_{ik} = e^{-t}(\delta_{ik} + \Delta t u_i v_k) \\ \langle r_i(t) \rangle = e^{-t}(v_i + \Delta t u_i). \end{cases} \tag{127}$$

Using Eqs. Eq. (81) and Eq. (127) we can write the correlation matrix $\mathbf{C}^g(t)$ as

$$C^g_{ij}(t) = \frac{g^2}{N} e^{-2t} \sum_{k,l} \int_0^t \mathrm{d}s_1 \big(\delta_{ik} + \Delta(t - s_1) u_i v_k\big)\big(v_l + \Delta s_1 u_l\big) \int_0^t \mathrm{d}s_2 \big(\delta_{jk} + \Delta(t - s_2) u_j v_k\big)\big(v_l + \Delta s_2 u_l\big). \tag{128}$$

By integrating over the variables $s_1$ and $s_2$ we find

$$\begin{aligned} C^g_{ij} = \frac{g^2}{N} e^{-2t} \sum_{k,l} & \delta_{ik}\delta_{jk} v_l^2 t^2 + \Delta\big(2\delta_{ik}\delta_{jk} v_l u_l + \delta_{ik} u_j v_k v_l^2 + \delta_{jk} u_i v_k v_l^2\big)\frac{t^3}{2} \\ & + \Delta^2\big(\delta_{ik}\delta_{jk} u_l^2 \delta_{ik} u_j u_l v_k v_l + \delta_{jk} u_i u_l v_k v_l + u_i u_j v_k^2 v_l^2\big)\frac{t^4}{4} \\ & + \Delta^2\big(\delta_{ik} u_j u_l v_k v_l + \delta_{jk} u_i u_l v_k v_l\big)\frac{t^4}{6} \\ & + \Delta^3\big(\delta_{ik} u_j u_l^2 v_k + \delta_{jk} u_i u_l^2 v_k + 2 u_i u_j u_l v_l v_k^2\big)\frac{t^5}{12} + \Delta^4 u_i u_j u_l^2 v_k^2 \frac{t^6}{36}. \end{aligned} \tag{129}$$

By projecting $\mathbf{C}^g$ on the direction $\mathbf{u}$, we find that only the order 1, $\Delta^2$ and $\Delta^4$ contribute:

$$\mathbf{u}^T \mathbf{C}^g \mathbf{u} = \sum_{i,j=1}^N u_i C_{ij}(t) u_j = \frac{g^2}{N} e^{-2t} \left( t^2 + \Delta^2 \frac{t^4}{2} + \Delta^4 \frac{t^6}{36} \right). \tag{130}$$

At the time of the peak amplification, i.e. for $t = t^* \simeq 1$, we recover Eq. (128).

To compute the correlation matrix relative to the input noise, we use Eqs. Eq. (127) and Eq. (81). As a result

$$\begin{aligned} C^\sigma_{ij}(t) = \sigma^2 \Bigg[ & \delta_{ij}\left(\frac{1}{2} - \frac{e^{-2t}}{2}\right) + \Delta(u_i v_j + u_j v_i)\left(\frac{1}{4} - \frac{e^{-2t}}{4} - \frac{te^{-2t}}{2}\right) \\ & + \Delta^2 u_i u_j ||\mathbf{v}||^2 \left(\frac{1}{4} - \frac{e^{-2t}}{4} - \frac{te^{-2t}}{2} - \frac{t^2 e^{-2t}}{2}\right)\Bigg]. \end{aligned} \tag{131}$$

By projecting $\mathbf{C}^\sigma$ evaluated at time $t = t^* = 1$ onto the readout $\mathbf{u}$, we find that only the order 1 and $\Delta^2$ contribute, resulting in Eq. (83).
