# Supplementary material for: Coding with transient trajectories in recurrent neural networks
Source: PLoS Comput Biol. 2020 Feb 13;16(2):e1007655. doi: 10.1371/journal.pcbi.1007655 (PMC7043794; doi:10.1371/journal.pcbi.1007655)
Supplement: S8 Text — (PDF) [file pcbi.1007655.s008.pdf]

# Coding with transient trajectories in recurrent neural networks

Giulio Bondanelli *[1], Srdjan Ostojic [1],

**1** Laboratoire de Neurosciences Cognitives et Computationelles, Département d'Études Cognitives, École Normale Supérieure, INSERM U960, PSL University, Paris, France

*giulio.bondanelli@ens.fr

## Supporting information

### S8 Text

In this section we study the dynamics of the norm of the component of the activity $\mathbf{r}(t)$ orthogonal to the plane defined by the two structure vectors $\mathbf{u}$ and $\mathbf{v}$. We focus on the case of uncorrelated structure vectors ($\rho \simeq 0$), so that the orthogonal component is given by $\mathbf{r}^{\perp}(t) \simeq \mathbf{r}(t) - \mathbf{u} \cdot \mathbf{r}(t) - \mathbf{v} \cdot \mathbf{r}(t)$. We assume that the condition for the strong amplification regime is satisfied (Eq. 55) and we set the external input to the vector $\mathbf{v}$, which is close to the amplified initial condition in absence of noise in the connectivity ($g = 0$).

To study the temporal evolution of $||\mathbf{r}^{\perp}||$, we project the dynamics onto a new orthonormal basis. We choose the first two basis vectors to be $\mathbf{u}$ and $\mathbf{v}$, while the choice of the remaining $N-2$ vectors is arbitrary, under the constraint that they form an orthonormal basis with $\mathbf{u}$ and $\mathbf{v}$. We call $\mathbf{T}$ the orthogonal matrix which contains the new basis vectors as columns. The rate model in Eq. (1) can be written in the new basis as

$$\dot{\tilde{\mathbf{r}}} = -\tilde{\mathbf{r}} + \tilde{\mathbf{J}}\tilde{\mathbf{r}} + \delta(t)\tilde{\mathbf{r}}_0, \tag{132}$$

where $\tilde{r} = (r_{\mathrm{u}}, r_{\mathrm{v}}, \mathbf{r}^{\perp})$, so that

$$||\mathbf{r}^{\perp}(t)|| = \sqrt{\tilde{r}_3^2(t) + ... + \tilde{r}_N^2(t)}. \tag{133}$$

The connectivity matrix in the new basis is

$$\tilde{\mathbf{J}} = \mathbf{T}^T\mathbf{J}\mathbf{T} \simeq \begin{pmatrix} \lambda & \Delta & \mathbf{J}_{\mathrm{u}\perp} \\ 0 & 0 & \mathbf{J}_{\mathrm{v}\perp} \\ \mathbf{J}_{\perp\mathrm{u}} & \mathbf{J}_{\perp\mathrm{v}} & \mathbf{J}_{\perp\perp} \end{pmatrix}, \tag{134}$$

where $\mathbf{J}_{\perp\mathrm{u}}$ and $\mathbf{J}_{\perp\mathrm{v}}$ are $(N-2)\times 1$ matrices, $\mathbf{J}_{\mathrm{u}\perp}$ and $\mathbf{J}_{\mathrm{v}\perp}$ are $1\times(N-2)$ matrices and $\mathbf{J}_{\perp\perp}$ is a $(N-2)\times(N-2)$ matrix. Since $\mathbf{T}$ and the connectivity noise $\boldsymbol{\chi}$ (see Eq. 70) are uncorrelated, the elements of these matrices have zero mean and variance equal to $g^2/N$. The elements $\tilde{\mathbf{J}}_{21}$ ans $\tilde{\mathbf{J}}_{22}$ are $O(1/\sqrt{N})$ and they have been set to zero in Eq. (134). By differentiating both sides of Eq. (133) and using Eq. (132) and Eq. (134), we can derive the equation for the dynamics of $||\mathbf{r}^{\perp}(t)||$, which reads:

$$\frac{\mathrm{d}||\mathbf{r}^{\perp}||}{\mathrm{d}t} = \frac{\mathbf{r}^{\perp T}(\mathbf{J}_{\perp\perp,S} - 1)\mathbf{r}^{\perp}}{||\mathbf{r}^{\perp}||^2}||\mathbf{r}^{\perp}|| + \frac{\mathbf{r}^{\perp} \cdot \mathbf{J}_{\perp\mathrm{v}}}{||\mathbf{r}^{\perp}||} r_{\mathrm{v}}(t) + \frac{\mathbf{r}^{\perp} \cdot \mathbf{J}_{\perp\mathrm{u}}}{||\mathbf{r}^{\perp}||} r_{\mathrm{u}}(t), \tag{135}$$

where $\mathbf{J}_{\perp\perp,S}$ denotes the symmetric part of $\mathbf{J}_{\perp\perp}$. Eq. (135) alone is not enough to solve for the dynamics of $||\mathbf{r}^{\perp}(t)||$, since it depends also on $\mathbf{r}^{\perp}(t)$. However we note that, for $t \gg 2/\Delta$ we have

$$\frac{\mathbf{r}^{\perp}}{||\mathbf{r}^{\perp}||} \simeq \mathbf{J}_{\perp\mathrm{u}}. \tag{136}$$

In fact, using Eq. (132) to compute the orthogonal activity for small times $\delta t$ we obtain

$$\mathbf{r}^{\perp}(\delta t) = \mathbf{J}_{\perp\mathrm{v}}\delta t + \frac{1}{2}(\Delta\mathbf{J}_{\perp\mathrm{u}} + \mathbf{J}_{\perp\perp}\mathbf{J}_{\perp\mathrm{v}})\delta t^2 + O(\delta t^3). \tag{137}$$

In the strong amplification regime (Eq. 55), for times $\delta t \gg 2/\Delta$ we have $\Delta||\mathbf{J}_{\perp\mathrm{u}}||\delta t^2 \gg 2||\mathbf{J}_{\perp\mathrm{v}}||\delta t + ||\mathbf{J}_{\perp\perp}\mathbf{J}_{\perp\mathrm{v}}||\delta t^2$, so that Eq. (136) holds up to corrections due to the input from the mode $\mathbf{v}$ and to the

feedback from $\mathbf{r}^\perp$ to itself. Numerical simulations confirm Eq. (136) and show that it holds also at larger times. The third term in Eq. (135) then becomes $gr_\mathrm{u}(t)$. Thus, neglecting the second term on the right hand side of Eq. (135), which decays exponentially, and cosidering the mean activity along $\mathbf{u}$ given by Eq. (78), we can write

$$\frac{\mathrm{d}||\mathbf{r}^\perp||}{\mathrm{d}t} = -\gamma(t)||\mathbf{r}^\perp|| + g\Delta t e^{-t}, \qquad \gamma(t) = -\frac{\mathbf{r}^{\perp T}(\mathbf{J}_{\perp\perp,S} - 1)\mathbf{r}^\perp}{||\mathbf{r}^\perp||^2}. \tag{138}$$

Note that at time $t = 0$ the elements of $\mathbf{r}^\perp$ and $\mathbf{J}_{\perp\perp,S}$ are uncorrelated, so that we have $\gamma(0) = 1$. Instead, the asymptotic dynamics in the orthogonal subspace is governed by the coupling matrix $\mathbf{J}_{\perp\perp}$ (see Eq. 134) so that the timescale of the decay of $||\mathbf{r}^\perp||$ is $1/(1 - \lambda_\mathrm{max}(\mathbf{J}_{\perp\perp}))$, with $\lambda_\mathrm{max}(\mathbf{J}_{\perp\perp}) = g - 1$. Therefore the asymptotic value of $\gamma(t)$ is given by $\gamma(+\infty) = 1 - g$. By solving Eq. (138) we obtain the expression for the dynamics of $||\mathbf{r}^\perp||$:

$$||\mathbf{r}(t)|| = g\Delta A\big(\gamma(g)\big), \quad A\big(\gamma(g)\big) = \int_0^t \mathrm{d}s\; s e^{-\int_s^t \gamma(z)\mathrm{d}z - s}. \tag{139}$$

Thus we find that, in presence of noise in the connectivity, the norm of the activity orthogonal to the $\mathbf{uv}$-plane scales linearly with $\Delta$.
