# Supplementary material for: Coding with transient trajectories in recurrent neural networks
Source: PLoS Comput Biol. 2020 Feb 13;16(2):e1007655. doi: 10.1371/journal.pcbi.1007655 (PMC7043794; doi:10.1371/journal.pcbi.1007655)
Supplement: S9 Text — (PDF) [file pcbi.1007655.s009.pdf]

Coding with transient trajectories in recurrent neural networks

Giulio Bondanelli [*1], Srdjan Ostojic [1],

1 Laboratoire de Neurosciences Cognitives et Computationelles, Département d'Études Cognitives, École Normale Supérieure, INSERM U960, PSL University, Paris, France

*giulio.bondanelli@ens.fr

Supporting information

S9 Text

The exponential of the sum of two matrices $\mathbf{A}$ and $\mathbf{B}$ can be factorized as

$$\exp(\mathbf{A}+\mathbf{B}) = \exp(\mathbf{A})\exp(\mathbf{B}) \tag{140}$$

only if $\mathbf{A}$ and $\mathbf{B}$ commute, i.e. if the commutator $[\mathbf{A},\mathbf{B}] = \mathbf{AB} - \mathbf{BA}$ is equal to zero. In the following we compute the mean and the variance of the commutator

$$C = [\Delta\mathbf{u}^{(1)}\mathbf{v}^{(1)T}, \Delta\mathbf{u}^{(2)}\mathbf{v}^{(2)T}] \tag{141}$$

and show that

$$\langle C_{ij}\rangle = 0, \qquad \langle C_{ij}^2\rangle \simeq \frac{2\Delta^4}{N^3} \tag{142}$$

The mean of C_{ij} is given by

$$\langle C_{ij}\rangle = \sum_{k=1}^{N}\left\langle \mathbf{u}_i^{(1)}\mathbf{v}_k^{(1)}\mathbf{u}_k^{(2)}\mathbf{v}_j^{(2)} - \mathbf{u}_i^{(2)}\mathbf{v}_k^{(2)}\mathbf{u}_k^{(1)}\mathbf{v}_j^{(1)}\right\rangle. \tag{143}$$

Since all the factors in the products on the right hand side are uncorrelated, we have $\langle C_{ij}\rangle = 0$. The variance of C_{ij} is given by

$$\begin{aligned}\langle C_{ij}^2\rangle = \sum_{k,l=1}^{N}\Big\langle &\mathbf{u}_i^{(1)}\mathbf{v}_k^{(1)}\mathbf{u}_k^{(2)}\mathbf{v}_j^{(2)}\,\mathbf{u}_i^{(1)}\mathbf{v}_l^{(1)}\mathbf{u}_l^{(2)}\mathbf{v}_j^{(2)} + \mathbf{u}_i^{(2)}\mathbf{v}_k^{(2)}\mathbf{u}_k^{(1)}\mathbf{v}_j^{(1)}\,\mathbf{u}_i^{(2)}\mathbf{v}_l^{(2)}\mathbf{u}_l^{(1)}\mathbf{v}_j^{(1)}\\ &- \mathbf{u}_i^{(1)}\mathbf{v}_k^{(1)}\mathbf{u}_k^{(2)}\mathbf{v}_j^{(2)}\mathbf{u}_i^{(2)}\mathbf{v}_l^{(2)}\mathbf{u}_l^{(1)}\mathbf{v}_j^{(1)} - \mathbf{u}_i^{(2)}\mathbf{v}_k^{(2)}\mathbf{u}_k^{(1)}\mathbf{v}_j^{(1)}\mathbf{u}_i^{(1)}\mathbf{v}_l^{(1)}\mathbf{u}_l^{(2)}\mathbf{v}_j^{(2)}\Big\rangle.\end{aligned} \tag{144}$$

The first term on the right hand side is thus given by

$$\begin{aligned}&\sum_{k,l=1}^{N}\left\langle \mathbf{u}_i^{(1)}\mathbf{v}_k^{(1)}\mathbf{u}_k^{(2)}\mathbf{v}_j^{(2)}\,\mathbf{u}_i^{(1)}\mathbf{v}_l^{(1)}\mathbf{u}_l^{(2)}\mathbf{v}_j^{(2)}\right\rangle = \sum_{k,l=1}^{N}\left\langle \mathbf{u}_i^{(1)2}\right\rangle\left\langle \mathbf{v}_j^{(2)2}\right\rangle\left\langle \mathbf{v}_k^{(1)}\mathbf{v}_l^{(1)}\right\rangle\left\langle \mathbf{u}_k^{(2)}\mathbf{u}_l^{(2)}\right\rangle\\ &= \sum_{k,l=1}^{N}\frac{1}{N^4}\delta_{kl} = \frac{1}{N^3}.\end{aligned} \tag{145}$$

Computing the second term yields the same result. For the third term we obtain

$$\begin{aligned}&\sum_{k,l=1}^{N}\left\langle \mathbf{u}_i^{(1)}\mathbf{v}_k^{(1)}\mathbf{u}_k^{(2)}\mathbf{v}_j^{(2)}\mathbf{u}_i^{(2)}\mathbf{v}_l^{(2)}\mathbf{u}_l^{(1)}\mathbf{v}_j^{(1)}\right\rangle = \sum_{k,l=1}^{N}\left\langle \mathbf{u}_i^{(1)}\mathbf{u}_l^{(1)}\right\rangle\left\langle \mathbf{v}_j^{(1)}\mathbf{v}_k^{(1)}\right\rangle\left\langle \mathbf{u}_k^{(2)}\mathbf{u}_i^{(2)}\right\rangle\left\langle \mathbf{v}_j^{(2)}\mathbf{v}_l^{(2)}\right\rangle\\ &= \sum_{k,l=1}^{N}\frac{1}{N^4}\delta_{il}\delta_{jk}\delta_{ki}\delta_{jl} = \frac{1}{N^4}\delta_{ij}.\end{aligned} \tag{146}$$

Using Eq. (145) and Eq. (146), we obtain Eq. (142). Thus, in the limit of large N we can write

$$\exp\left(t(\Delta\mathbf{u}^{(1)}\mathbf{v}^{(1)T} + \Delta\mathbf{u}^{2)}\mathbf{v}^{(2)T} - \mathbf{I})\right) = e^{-t}\exp\left(t(\Delta\mathbf{u}^{(1)}\mathbf{v}^{(1)T})\right)\exp\left(t(\Delta\mathbf{u}^{(2)}\mathbf{v}^{(2)T})\right) \tag{147}$$

and recover Eq. (90).
