# Supplementary figures and images for: Coding with transient trajectories in recurrent neural networks
Source: PLoS Comput Biol. 2020 Feb 13;16(2):e1007655. doi: 10.1371/journal.pcbi.1007655 (PMC7043794; doi:10.1371/journal.pcbi.1007655)

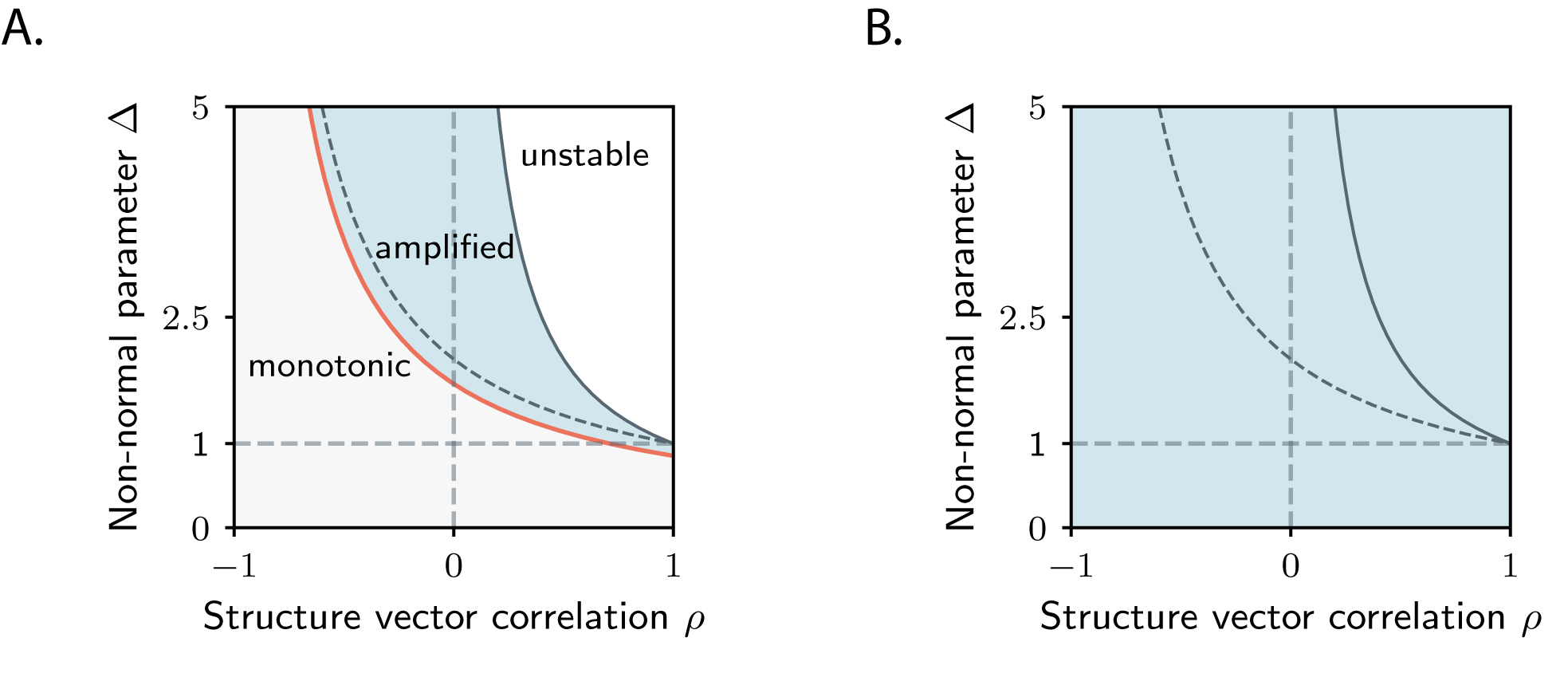

Supplement: S1 Fig — A. g<1/2. The red line indicates the boundary between the monotonic and amplified parameter regions for g = 0.5. The grey dashed line corresponds to the case g = 0. B. g>1/2. The dynamics are amplified regardless of the values of the parameters Δ and ρ. (TIF) [file pcbi.1007655.s010.tif]

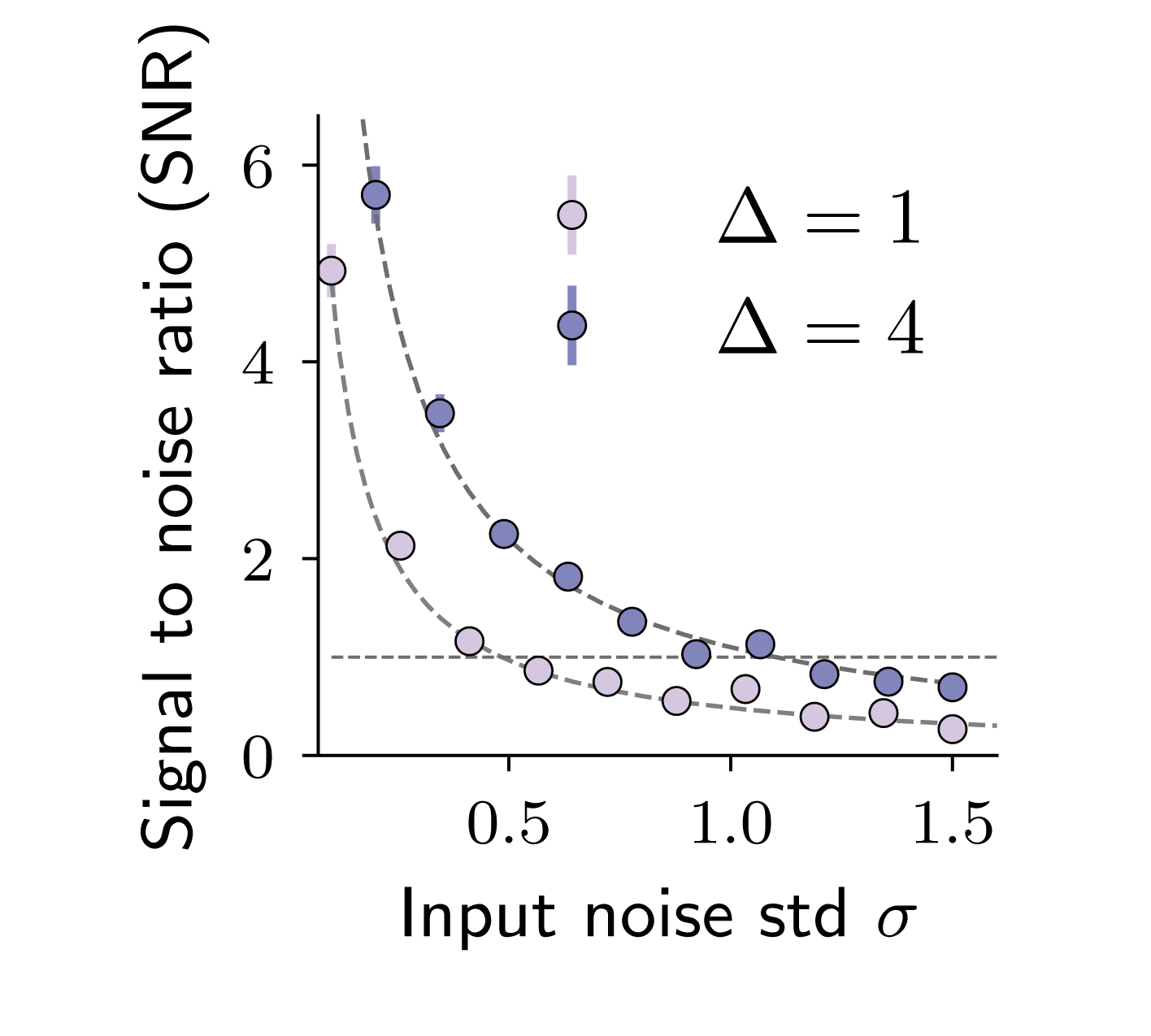

Supplement: S2 Fig — Signal-to-noise ratio of the readout as a function of the standard deviation of the input noise σ for two values of the non-normal parameter Δ. Non-amplified dynamics (Δ = 1) are less robust to noise than amplified dynamics (Δ = 4). Dashed lines correspond to the theoretical values (Eq 85). In simulations, N = 1000. Errorbars represent the standard deviation of the mean over 200 realizations of the connectivity matrix. (TIF) [file pcbi.1007655.s011.tif]
