# Supplementary material for: Coding with transient trajectories in recurrent neural networks
Source: PLoS Comput Biol. 2020 Feb 13;16(2):e1007655. doi: 10.1371/journal.pcbi.1007655 (PMC7043794; doi:10.1371/journal.pcbi.1007655)
Supplement: Attachment — Submitted filename: response.pdf [file pcbi.1007655.s012.pdf]

We would like to thank the Reviewers for their nice words and thorough comments on the manuscript. We report below our responses to each of the comments.

Reviewer #1:

Comments:
1. Lines 93-94 (and also lines 162): The language needs to be refined/made more precise. It's, strictly speaking, meaningless to talk about identifying "different inputs giving rise to amplified trajectories" or estimating "their numbers", or asking "… how many inputs are amplified?", since the input space (and its subspace with those properties) is a continuum, or more precisely a vector space with infinite members. You should rather say "identity the input subspace giving rise to amplified trajectories" and "estimating the dimensionality of this subspace", and/or "how many orthogonal inputs [or input patterns] are amplified?".

We thank the reviewer for pointing this out, we have corrected this in the manuscript.

2. Lines 126-127: you say "… except in the case of 126 one-dimensional dynamics or symmetric connectivity matrices." The exception is not limited to symmetric matrices and should cover all normal J's, so this phrase should be corrected. (E.g. consider anti-symmetric J's which have imaginary eigenvalues: in that case the symmetric part is 0 and all its eigenvalues are 0, so the second condition is 0 >1, while because eigenvalues of J are imaginary the first condition is 0 < 1, and the two are inconsistent.)

The exception indeed holds for all normal matrices, including symmetric, anti-symmetric and orthogonal matrices. We have corrected this in the manuscript.

3. Line 210: you end the sentence by "… when the connectivity J is Gaussian.", but the correct statement is "… when the connectivity matrix J is a random matrix with independent and identically distributed elements." Gaussianity is not key (due to universality theorems on the circle and semicircular laws), but independence is key: so conversely a (symmetric) gaussian matrix with nontrivial covariances across elements need not give rise to the circle (semicircular) law.

We thank the reviewer for pointing this out, we have corrected this in the manuscript.

4. Caption of Fig. 4: Change the caption for panel C to "C. Illustration of the dynamics elicited by the three inputs, R_1, R_2 and R_100 (show in different rows), as in B."

Corrected in the caption.

5. Lines 228 and 231: you need to define what you mean by minimal connectivity. It seems that you mean a matrix with minimal rank. So try to justify why that notion of minimality is relevant to neuroscience.

Indeed we mean minimal rank, we have now corrected this, and mention the ubiquity of low-rank networks in neuroscience.

6. Lines 280-292: the writing has to be made accurate regarding the "orthogonality" of different pattens. If the u_p/v_p vectors pairs for different p's are literally mutually orthogonal (as the current text suggests) then there will be exactly zero interference between different rank-one terms (i.e. different patterns), and fluctuations of the activity along a readout u_p caused

by other patterns will be exactly 0, and not just O(\sqrt{P}/N). In this case the "capacity" would be N/2. But reading the Methods section it becomes clear that the authors are implicitly talking about a connectivity with the rank-P form with the different u's and v's sampled from a random ensemble of vectors with independently distributed components, such that different vectors are only approximately orthogonal with high probability (when N is large), and not literally orthogonal as the main text in lines 280-290 suggests. So this should be corrected.

We have now made explicit that our derivation holds when the elements of the vector u_i and v_i are independently sampled from a random distribution (e.g. Gaussian). When all the vectors u_i and v_i are mutually orthogonal the capacity is indeed equal to N/2.

7. Lines 27-28: replace "… but sufficient conditions for such amplification were not given." with "… but general sufficient conditions for such amplification were not given."

Corrected in the manuscript.

8. Line 53: I would add the following at the end of this sentence (first sentence of "Monotonic vs Amplified Transient Trajectories"): "… in the autonomous network with I(t) = 0."

We corrected with "…in the autonomous network". In the autonomous network the input is given by I(t)=delta(t).

9. Lines 740, 749 and 763: fix the broken (appearing as ?) references.

We apologize about this, references have been fixed.

10. The brackets (signifying averaging) around dr_i on the left hand side should be removed.

Corrected in the manuscript.

11. In equation 80 (lines 782-783): the sum over the index l is only needed for the first term and the second term (the one involving \eta_k) should not be summed over l.

We thank the reviewer for pointing this out, we have corrected this in the manuscript.

12. Line 817: "randomly distributed" should be replaced with "randomly and independently distributed".

Corrected in the manuscript.

13. Line 343: "straightforward" doesn't have a hyphen.

Corrected in the manuscript.

14. Figure 6: In panel E, the maximum readout value for P/N ~ 0.02 seems to be significantly higher than the maximum of the red curve in panel D. Why?

Thank you for pointing this out. We must have wrongly used two different realizations of the connectivity J, which led to the inconsistency that you pointed out. This has been corrected in the figure, where panel E and D are now consistent.

15. Figure 2: in panel B, it would be helpful to also include a line for \tau>1, corresponding to max Re \lambda(J) >0.

The line corresponding to tau=1 has been added.

16. Figure 2: panel C would be more informative if it is turned into a heat-map of \sigma_1^*, with the two phases (monotonic and amplified) and their boundary indicated/drawn on top of the heat-map.

Panel C has been turned into a heat map.

17. In caption of Fig. 4, panel B: replace "… at time t_* in pannel [sic.] A" with "… at time t_* indicated by the dashed vertical line in panel A".

Corrected in the caption.

18. Fig 4 C, last row, middle column: unlike in the top and middle row of panel C, the labels L_100^* and R_100^* are missing here.

Corrected in the figure.

19. Line 544: replace "symmetrically arranged along the imaginary dimension." with "symmetrically arranged on either side of the real axis."

Corrected in the manuscript.

,

Reviewer #2:

*1) I had the intuition that strong amplification required not only non-normality (lack of orthogonality of the eigenvectors of J), but also a **difference** between their corresponding eigenvalues. In this case, initial conditions loading on several non-orthogonal eigenvectors (i.e., those involving 'cancellations') align initially with the slowest decaying modes (this is the period of growth), and eventually decay. Is this intuition incorrect in general, or is this picture somehow hidden in the properties of J_s?*

We share this intuition with the reviewer, but are not able to prove it beyond simple 2x2 cases.
As the Editor has pointed out, an initial condition given by the linear combination of eigenvectors whose eigenvalues are all the same will undergo a (one-dimensional) decay to the zero fixed point. However, if the initial condition is given by the linear combination of eigenvectors with slightly different eigenvalues we do not expect amplified dynamics (in terms of the norm ||r(t)||), since the decay along all the eigenvectors would have similar timescales. Amplified dynamics must therefore depend on an interplay between the difference in eigenvalues and the relative overlap between the eigenvectors. This can be easily seen for the rank-1 network structure studied in the manuscript. If we assume rho not zero, we have two eigenvectors in the u-v plane: the first eigenvector is u with eigenvalue lambda; the second is the vector perpendicular to v, with eigenvalue 0. Therefore the difference between the eigenvalues is given by lambda. Using Eq.(59) we can write the maximum amplification of the system as A(lambda, rho) = (1-lambda)^(1/lambda-1)*lambda/rho. For fixed rho, A is a monotonic and increasing function of lambda (remembering that sgn(rho)=sgn(lambda)). This indicates that, for the rank-1 network, a dif-

ference in the eigenvalues on the u-v plane is necessary to have amplification and that the maximum amplification decreases as 1/sqrt(1-O^2) when the overlap O between the two eigenvectors decreases (and therefore “approaches” a normal network). While the connection between amplification, difference between eigenvalues and overlap between eigenvectors, may be possible to derive for simple cases, it might be hard to derive for general network structures. Ideally this connection would involve two different levels of description. The first level is a global one, describing if the network is in the amplified regime or not. In our framework this property is determined by the numerical abscissa; an analogous measures based on eigenvalues and eigenvectors of J would need to take into account all the interactions (to which order?) between all the eigenvalues and eigenvectors of J (an upper bound on the maximum amplification can be established based on the condition number of the eigenvector matrix of J (Eq.14.5 in “Spectra and pseudospectra”, Trefethen and Embree, 2005), but is in general a loose bound). The second level is a local one, concerning the specific inputs that can be amplified by the network. In our framework, a natural set of amplified (at t=0) inputs is the eigenvectors of J_S corresponding to eigenvalues larger than 1. A connection at this level would need to describe on which eigenvectors of J the amplified inputs (e.g. the eigenvectors of J_S) are loaded on and the mutual relationships between those eigenvectors of J (and the corresponding eigenvalues). Both of these connections seem hard to make in a general network, because (1) few results are available for eigenvectors (compared to eigenvalues) in random matrix theory and (2) the relationship between the eigenvectors of J and the ones of J_S is even more difficult to characterise for a general network. How the amplification depends on the relationship between eigenvalues and eigenvectors of the connectivity J itself is therefore exciting and fundamental question that needs to be addressed to understand the dynamics of non-normal networks.

2) Transient amplification (TA) is usually studied in connection to specific initial conditions from which amplification happens. The ‘transient’ is then purely generated by the recurrent connectivity. From the point of view of neuroscience, I think it would be interesting to include some comments in the discussion about the kinds of experimental or real-life situations where this may be expected to occur or not. For instance, TA has been related to preparatory states before movement. How about onset sensory responses in cortex? How about time-varying inputs (which actually are the ethological norm)? The discussion on this would benefit from the recognition that, although linear models provide interesting approximations, the real networks have significant non-linearities, even at the firing rate level… (I say this because if everything is purely linear, there is perfect superposition in time and time-varying input is not particularly interesting...)

Thank you, we added a discussion paragraph on these considerations. We discuss how transient amplification (with autonomous dynamics) has been applied to the study of neural activity in motor and sensory areas. We point out how the assumption of autonomous dynamics is nonetheless restrictive when more complex stimuli are considered. However, though of paramount importance in neuroscience, the behaviour of non-normal networks in response to temporal stimuli has not to our knowledge been investigated yet (except for periodic forcing inputs in Farrell, Ioannou ”Generalized stability theory. Part I: Autonomous operators”, 1996).
We discuss non-linearities in the last paragraph of the Discussion.

Reviewer #3: # Summary and main comments

1. Perhaps my main comment concerns the old debate on matrix nonnormality, and how it is inherently difficult to characterise a concept that has to do with the geometry of a matrix'

eigenbasis using a single scalar number (here, the numerical abscissa). In this paper, the distinction is made between networks in which the activity vector can grow transiently in response to an appropriately chosen input; and those that simply can't, regardless of the input. The authors derive a diagnostic criterion based on the numerical abscissa; while this is entirely valid (given the definition of "amplifying vs non-amplifying" networks outlined above), it is nevertheless an asymptotic criterion which only concerns the slope of the norm $\| r(t)\|$ of the response at small times ($t=0^+$) and therefore _in principle_ provides little insight about how much $\| r(t) \|$ will grow and for how long. To be fair, the authors go on discussing an analysis of the induced 2-norm of the propagator e^{tA}, which gives the full description of transients, but – as they acknowledge – whose SVD can only be computed numerically in most cases. There is a whole chapter in Trefethen & Embree ("Spectra and pseudospectra"; chap. 4 on transients) which discusses alternative summary scalar measures that are much better indications of expected transient magnitude / durations (i.e. provide useful bounds on transient size & timing). The pseudospectrum and associated Kreiss theorems should probably be mentioned.

So I suggest the authors discuss these issues briefly in the intro / discussion. Perhaps a useful place to start is the 2x2 example they have already studied in depth, for which it's not too difficult to find corner cases where the max amplification is bounded whereas the numerical abscissa isn't. E.g. the peak amplification for W = [1; -a]; [1; -a]] is upper bounded by $\sqrt{2}$ (it tends to $\sqrt{2}$ as $a \to \infty$); yet the numerical abscissa (spectral abscissa of $W+W^T-2I$) grows linearly in a ($\approx (\sqrt(2)-1) a$ (the peak of amplification occurs earlier and earlier, too).

We fully agree with the reviewer. The value of the numerical abscissa determines if a network can or cannot amplify external inputs, but it does not in general provide a measure of the maximum amplification of the norm ||r(t)|| over all times t. We added this consideration in a discussion paragraph, pointing out the existence of more apt measures of the maximum amplification in non-normal systems (pseudospectra and Kreiss Matrix Theorem).
We now illustrate the fact that the value numerical abscissa cannot in general be directly related to the maximum amplification at the end of the section “Maximum amplification of the system” (p.24). For fixed rho<0, increasing Delta (thus increasing the numerical abscissa) leads to a saturation of the maximum amplification to a value equal to 1/|rho|. The 2x2 example that you suggested can be mapped to the third case when a>>1, so that rho=-1/sqrt(2).

2. Going back to the "coding" viewpoint, adopted in the introduction: for the type of deterministic Markovian dynamics considered here, future states are entirely determined by the current one – the whole trajectory is entirely determined by the initial condition; so for deterministic dynamics, there can be no advantage of eliciting fancy transients: there is just as much information about the stimulus at the peak of the transient as there was at $t=0$. The advantage of amplification for coding become apparent when noise is taken into consideration – but then, it is important to distinguish between process noise (which tends to get amplified together with signal) and observation noise (which doesn't). Nonnormal transients don't help much with process noise (though that depends on its geometry), but can hugely increase the mutual information between stimulus and r(t) in cases when output (observation) noise is strong; having signal rise above noise is a big win. I would recommend discussing this briefly, too.

Thank you for the interesting observation. We added this consideration in a discussion paragraph. At the end the section "Robustness of the readout activity" (p.29) we show that the critical values of the input noise sigma (above which SNR<1) along the readout (u) and the initial condition (v) are comparable (respectively sigma_c=1.17 and sigma_c0=1), with a small gain of 17% for strongly amplified networks. We point out that, even if the gain in SNR is not large, having an amplified system may be desirable to have a stable SNR across the transient dynamics (in particular at the initial condition and readout).

# Minor comments:

1. line 30:
> *This results leads to a simple distinction between two classes of networks: networks in which all inputs lead to weak, decaying transients, and networks in which specific inputs elicit strongly amplified transient responses.*

Cf main comment (1) above: this is a little too strong, as the class of networks of the latter type also contains networks that amplify very weakly.

Corrected in the manuscript.

2. line 71:
> *necessarily implies that the firing rate of at least one neuron shows a transient increase before decaying to baseline.*

increase, or decrease (but can always be turned into an increase by reversing the sign of the initial condition of course)

Corrected in the manuscript.

3. line 177:
> *Note that for a normal matrix, the left and right singular vectors $R(t)_k$ and $L(t)_k$ are identical, and the singular values are equal to the eigenvalues [...]*

corner case: e.g. skew symmetric (hence normal) matrix W has complex eigenvalues, whereas singular values are real

Corrected in the manuscript.

4. regarding the margin:
> *To eliminate the trajectories with very short amplification, one can further constrain the slopes to be larger than a margin ϵ*

again, this asymptotic result concerns the slope at time t=0; even with a margin, this seems to give no formal guarantee on the amount of amplification that can follow

Corrected in the manuscript.

5. line 228:
> *Specifically, we wish to determine the minimal connectivity that transiently transforms a fixed, arbitrary input r_0 into a fixed, arbitrary output w, through two-dimensional dynamics.*

Considerations of time unclear here → transform r_0 into w _at some point_ during the transient?

Added in the manuscript: we want that the initial condition r_0 is mapped onto a vector w at the peak amplification (time of maximum norm $||r(t)||$).

6. reference missing on line 405

Fixed.

7. reference to figure missing on line 808.

Fixed.
