# Supplementary material for: Coding with transient trajectories in recurrent neural networks
Source: PLoS Comput Biol. 2020 Feb 13;16(2):e1007655. doi: 10.1371/journal.pcbi.1007655 (PMC7043794; doi:10.1371/journal.pcbi.1007655)
Supplement: Attachment — Submitted filename: response_2.pdf [file pcbi.1007655.s013.pdf]

We would like to thank the Reviewers for supporting the publication of the manuscript and the Editor for his in-depth comments on the manuscript. We report below our responses to each of the comments.

*Abstract: "and networks in which specific inputs elicit strongly amplified transient responses and are mapped onto orthogonal output states during the dynamics". Here you are talking about the whole class of networks that show initial amplification, which may not be strongly amplified and need not be mapped onto orthogonal states, so this should be changed, e.g. to "and networks in which specific inputs elicit amplified responses during the dynamics". You could if you want restore the orthogonal concept by inserting 'orthogonal' in "We then build minimal, low-rank networks ... mapping a specific input onto a specific *orthogonal* output state."*

We thank the Editor for pointing this out. This has been corrected in the manuscript as suggested by the Editor.

Line 62: monotonic decay "exploring essentially a single dimension" -- this isn't true, the monotonic decay could curve or spiral through an arbitrarily high-dimensional space. So I suggest cutting this phrase. 'or transiently move away from it by following a rotation': similarly, this need not be a rotation -- for example if in 2D you have two eigenvectors both nearly vertical, and you start with a small horizontal initial condition, and eigenvalues are real and of very different magnitudes, then the trajectory will go up toward the eigenvector with the larger eigenvalue and then decay down along that eigenvector, which is nearly an up and down motion with only a slight rotational component. So I'd suggest cutting "by following a rotation".

We fully agree with the Editor. While this was an empirical observation in the case of random Gaussian connectivity matrices it needs not be the case in general. We corrected this in the manuscript.

Line 72: "the firing rate of at least one neuron shows a transient increase (or decrease) before decaying to baseline" -- 'increase or decrease' includes pretty much everything, so this is more or less a tautology. I think what you want to state is that at least one r_i shows a transient increase in its absolute value.
You've also gotten yourself into a bit of trouble since you are calling the r_i's the deviation of the firing rate from the fixed point, so you cannot simply refer to them as 'the firing rates', i.e. you can't say 'at least one firing rate increases in its absolute value'. You could get yourself out of this by, under Eq. 1, saying that for simplicity you will refer to the r_i's as the firing rates, in which case you would want to say 'before decaying to zero' instead of 'before decaying to the baseline'.

We agree with the Editor. This has been corrected in the manuscript.

Lines 144-147: "two interacting excitatory-inhibitory populations" is confusing, it sounds like 4 populations, two excitatory and two inhibitory. You could just say 'an excitatory and an inhibitory population'.
"our criterion states that the excitatory feedback needs to be (approximately) larger than unity in order to achieve transient amplification (Fig. 2 and Methods)". 'Methods' should instead be Appendix F. But also, this statement isn't correct -- it's only correct when the network is of the form {{w,-k w},{w,-k w}} with k=1+\epsilon for \epsilon small. It is certainly not true for the general case of one excitatory and one inhibitory population. The same problem is in the leg-

end of Figure 2, which again states this same criterion, now the form of the matrix is specified but you haven't noted the restriction on k.

We thank the Editor for pointing this out. The criterion w>1 indeed approximately holds for k=1+epsilon. We corrected this in the main text and in the caption of Figure 2.

Lines 315 and 323: I think you want to cite citation [37], not [38]

We apologise about this, we indeed meant reference [37]. This has been corrected in the manuscript.

Lines 378-380: "the inhibition-dominated regime, which as we show approximately corresponds to the class of unit-rank E-I networks satisfying the general criterion for transient amplification." This isn't true -- if you assume the form {{w,-k w},{w,-k w}}, you can get transient amplification in a stable network either with k<1 or k>1.
(Mathematica tells me: If k<1, the criterion is (2 (-1 + k))/(1 + k)^2 + 2 Sqrt[2] Sqrt[(1 + k^2)/(1 + k)^4] < w < -(1/(-1 + k)); while if k>1, the criterion is w > (2 (-1 + k))/(1 + k)^2 + 2 Sqrt[2] Sqrt[(1 + k^2)/(1 + k)^4].)

We thank the Editor for addressing this aspect. We fully agree with the Editor that a stable EI network of the form {{w,-k w},{w,-k w}} can be transiently amplified either with k>1 or 0<k<1. We have corrected this in the manuscript (Discussion and Appendix F) and updated Fig 2C to show the region k<1.

*Methods, Eq. 30: You should note that, *for J stable*, Eq 30 is the criterion for Eq. 28 to be >1, and that the stability condition that Det(J-1)>0 is precisely the condition that the argument of the square root in (30) is positive, so that \Delta_c is real. Without the condition that J is stable, the criterion for Eq 28 > 1 is more complicated.*

We thank the Editor for pointing this out. We clarified this in the manuscript, where we specified that Eq.30 holds under the assumption that J is stable.

Appendix F, line 1012 "recovers the results from (32), showing that the system is amplified if the excitatory strength w is (approximately) larger than one." In (32), we showed something different: that for an initial condition of r_E>0, r_I=0, r_E (not |r|) shows transient amplification if and only if w>1, for any value of k. What you are showing is that there will be some initial condition for which |r| will show transient amplification if k=1+\epsilon and w>1-\epsilon^2/4 for \epsilon<<1. These are very different.

We apologise to the Editor. Our result based on the norm |r| is indeed different from the result derived in Murphy and Miller, which takes into account the firing rate r_E. We corrected this in the manuscript.
